# How Mechanical Forces Change the Human Endometrium during the Menstrual Cycle in Preparation for Embryo Implantation

**DOI:** 10.3390/cells10082008

**Published:** 2021-08-06

**Authors:** Anna K. Sternberg, Volker U. Buck, Irmgard Classen-Linke, Rudolf E. Leube

**Affiliations:** Institute of Molecular and Cellular Anatomy, RWTH Aachen University, Wendlingweg 2, 52074 Aachen, Germany; asternberg@ukaachen.de (A.K.S.); vbuck@ukaachen.de (V.U.B.); iclassen-linke@ukaachen.de (I.C.-L.)

**Keywords:** mechanobiology, endometrial epithelium, embryo, endometrial crosstalk, adhesion, cytoskeleton, mechanosensitive ion channels, extracellular matrix, trophoblast, decidua

## Abstract

The human endometrium is characterized by exceptional plasticity, as evidenced by rapid growth and differentiation during the menstrual cycle and fast tissue remodeling during early pregnancy. Past work has rarely addressed the role of cellular mechanics in these processes. It is becoming increasingly clear that sensing and responding to mechanical forces are as significant for cell behavior as biochemical signaling. Here, we provide an overview of experimental evidence and concepts that illustrate how mechanical forces influence endometrial cell behavior during the hormone-driven menstrual cycle and prepare the endometrium for embryo implantation. Given the fundamental species differences during implantation, we restrict the review to the human situation. Novel technologies and devices such as 3D multifrequency magnetic resonance elastography, atomic force microscopy, organ-on-a-chip microfluidic systems, stem-cell-derived organoid formation, and complex 3D co-culture systems have propelled the understanding how endometrial receptivity and blastocyst implantation are regulated in the human uterus. Accumulating evidence has shown that junctional adhesion, cytoskeletal rearrangement, and extracellular matrix stiffness affect the local force balance that regulates endometrial differentiation and blastocyst invasion. A focus of this review is on the hormonal regulation of endometrial epithelial cell mechanics. We discuss potential implications for embryo implantation.

## 1. Introduction

**Figure 1:**
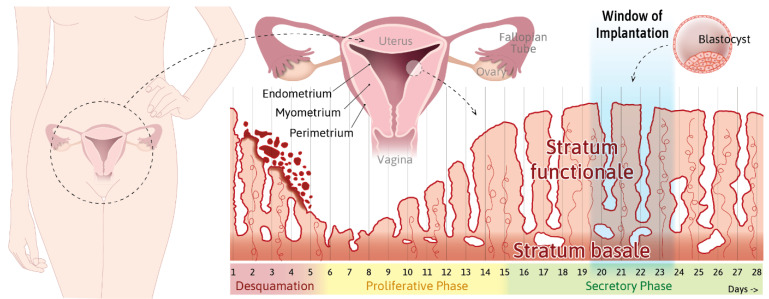
The plasticity and contractility of the human uterus require the constant adjustment of the endometrium to prepare and support embryo implantation and enable subsequent embryo development. The scheme highlights the multilayered wall structure of the uterus and menstrual cycle-dependent endometrial changes. The uterus is positioned in the pelvis below the peritoneal cavity. The inverted cone-shaped organ is connected through the Fallopian tube to the ovaries and opens into the vagina. It consists of three major layers: the outer connective tissue-rich perimetrium, the smooth muscle-containing contractile myometrium, and the inner endometrium. The endometrium can be further subdivided into the permanent stratum basale and the transitory stratum functionale, which is shed during desquamation and rebuilt during the proliferative phase, with changes in differentiation during the secretory phase to prepare for blastocyst implantation during the window of implantation. For further details, see Info Boxes 1 and 2 (Appendix A).

**Figure 2:**
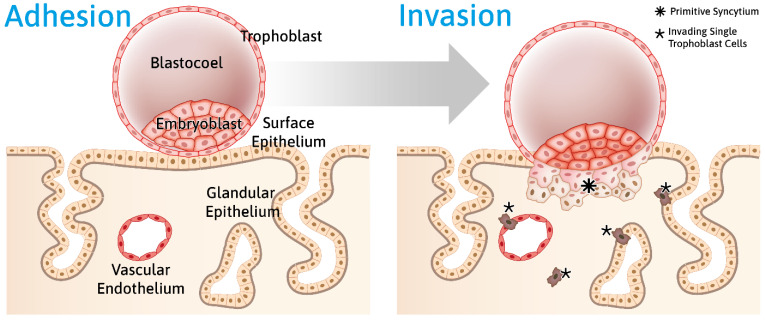
Schematic representation of blastocyst adhesion to the endometrial epithelium and subsequent trophoblast invasion into the endometrium. The developing blastocyst—consisting of the fluid-filled blastocoel, the embryoblast encompassing the inner cell mass, and the outer trophoblast cell layer—adheres to the uterine surface epithelium. Adhesion induces processes in both the embryonic blastocyst and the maternal epithelial and stromal cell layers, which result in the transmigration of the trophoblast through the epithelial layer and the invasion of the endometrial stromal compartment. Subsequently, specialized single trophoblast cells, the extravillous trophoblasts, invade the decidua and erode endothelia and glandular epithelia from their respective basal sides. For further details, see Info Box 3 (Appendix A).

**Figure 3:**
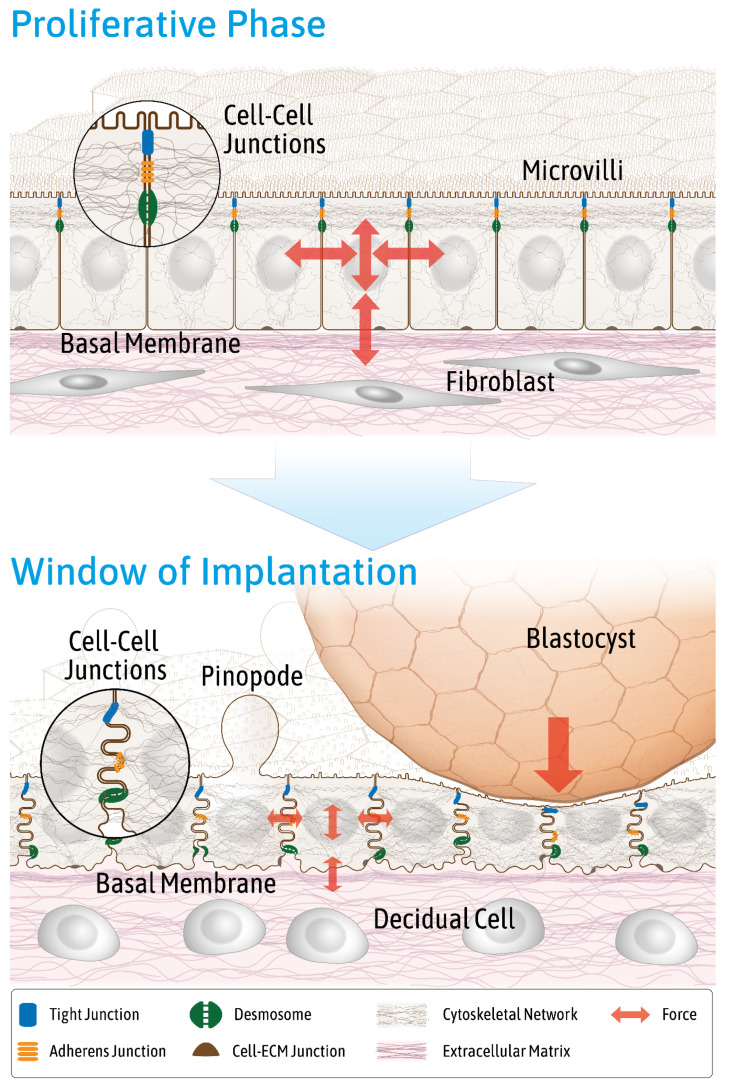
The schemes highlight endometrial changes affecting endometrial biomechanics during the menstrual cycle in preparation for blastocyst adhesion and invasion. Key features of the endometrium during the proliferative phase are depicted at the top, and key features during the window of implantation are depicted at the bottom. Note the differences in the endometrial epithelial cell layer including cell height, apical surface specializations (e.g., microvilli and pinopodes), cytoskeletal organization, nuclear morphology and position, distribution of lateral cell–cell junctions, and basolateral plasma membrane invaginations. Additionally note the changes in the connective tissue compartment with a reduced fiber content and altered fibroblast morphology during the window of implantation, thus providing a different biomechanical environment for the epithelial cell layer. The adhering blastocyst impacts the epithelium from the apical side. The red arrows indicate different types of forces that act in all spatial directions within and on the endometrial epithelium. They include shear stress from the uterine lumen, hydrostatic pressure from the adhering blastocyst, tensile forces within the epithelium, traction forces resulting from the epithelial–stromal interphase, and intracellular contractile forces. Epithelial viscoelasticity and extracellular matrix stiffness have impacts on force distribution.

According to the World Health Organization, one in ten couples is dependent on assisted reproductive technology [1,2]. The causes for infertility are manifold and include insufficient sperm production, hormonal imbalances, and stress [3,4]. Aspects that have received little attention are hormone-dependent changes in the biomechanical properties of the uterine endometrium that allow it to become receptive for embryo implantation [5]. The endometrium is the innermost layer of the uterine wall (Appendix A; Figure 1). It is adjacent to the contractile myometrium and the outermost perimetrium, which abuts the pelvic connective tissue and extends into the peritoneal cavity. The endometrium consists of surface epithelium that is connected to the uterine glands, which are embedded in loose connective tissue. Major structural changes occur in the endometrium during the ~28 day menstrual cycle (Appendix A; Figure 1 and Figure 2), whereby the upper part, i.e., the functional layer or stratum functionale of the endometrium, is completely shed during desquamation, rebuilt during the ensuing proliferative phase, and restructured during the secretory phase. Estrogen is the major stimulant during the proliferative phase, and it is coupled to glandular, connective tissue, and blood vessel growth, whereas progesterone is the major stimulant during the secretory phase. The secretory phase is characterized by glandular secretion coincident with altered permeability, adhesion, and surface receptor expression in the epithelial compartment, as well as with the decidualization of the connective tissue compartment. Decidualization involves drastic changes in the extracellular matrix and the partial mesenchymal–epithelial transition of resident fibroblasts, which together result in tissue softening [6]. Endometrial proliferation and differentiation prepare the uterine wall for embryo implantation. We argue that these preparatory changes alter the mechanical properties of the endometrium in such a way that they enhance the adhesion of the blastocyst and its migration through the epithelial surface layer into the underlying connective tissue compartment (Appendix A; Figure 3). Evidence for this concept is the observation that implantation is only possible during a short period of the secretory phase. This approximately two-to-four day receptive period is referred to as the window of implantation. 

The preparation of the endometrial epithelium and, even more so, the attachment and invasion of the developing blastocyst require a tight and coordinated force balance between maternal and embryonic components. This view is one of the basic tenets of the expanding field of mechanobiology (Appendix A, Glossary). Mechanobiology is based on the understanding that genetic programs are modulated by mechanical stimuli of the environment. The modulatory effects are balanced by complex feedback mechanisms and result in coordinated emergent tissue behavior. They require mechanosensing and mechanotransmission through specialized molecules (such as channels and extensible polypeptide domains) and structures (adhesion sites), as well as mechanotransduction through signaling pathways involving biochemical signals to elicit cellular responses (e.g., reorganization of the cytoskeleton, contraction, and proliferation), which together lead to a new force balance. Mechanical forces thereby contribute to tissue homeostasis and are involved in development and repair. The dysfunction of these mechanisms and feedback loops results in disease and tumorigenesis.

In the following paragraphs, we highlight aspects of the mechanophysical plasticity of the human endometrium during the menstrual cycle in preparation for embryo implantation. 

## 2. Mechanical Properties of the Endometrium Change during the Menstrual Cycle

To study menstrual cycle-dependent mechanical properties of the endometrium in vivo, three-dimensional multifrequency magnetic resonance elastography combined with multifrequency dual elasto–visco inversion has been used [7]. This is a novel, non-invasive approach to study the haptic material properties of tissues in vivo and allows one to measure the response of viscoelastic material to vibration, which is described as complex shear modulus |G *| [7]. The |G *| of the endometrium was found to be higher during the proliferative phase (3.34 ± 0.42 kPa) than during the early secretory phase (1.97 ± 0.34 kPa) in healthy volunteers [8]. Whether these differences reflect overall differences of the entire endometrium or differences between the functional and basal endometrial layer cannot be decided. However, we have assumed that the very loose connective tissue surrounding the extensive glands of the stratum functionale contributes to the increased softness during the secretory phase.

An important aspect of the steroid hormone-dependent mechanical properties of the endometrium are the known menstrual cycle-dependent changes in basement membrane composition and the overall changes in extracellular matrix composition. Stromal edema and reduced fibrous components are hallmark features of the connective tissue compartment during the mid secretory phase [9]. The general belief is that tissue-loosening aids implantation. An immunohistochemical study of the human endometrium revealed drastic changes in immunoreactivity for various extracellular matrix components, most notably including the basement components collagen type IV and laminin [10]. Another study [11] reported the detection of collagen type IV, fibronectin, and laminin during the secretory phase in the endometrial stroma of normal fertile women, as well as consistently reduced or absent levels of these polypeptides in women suffering from unexplained infertility. Of relevance may be the observed transient reduction of laminin and fibronectin around the window of implantation. Tanaka et al. [12] immunostained endometrial biopsies for collagen IV and laminin, and they found reduced laminin in the basement membrane of the endometrial surface epithelium and, to a lesser extent, in the basement membrane of the glandular epithelium during the secretory phase. In contrast to the other menstrual cycle phases, collagen IV was slightly decreased in the basement membrane of both luminal and glandular endometrial epithelial cells during the late proliferative phase, indicating an estrogen-dependent regulation [12]. Taken together, the reduction of the basement membrane component collagen IV (which is highly resistant to mechanical forces because of its cross-linked trimeric subunits [13,14]) and the reduction of laminin weaken the basement membrane of the endometrial epithelium. This may help extravillous trophoblast cells invade endometrial glands to provide histiotrophic nourishment for the embryo.

## 3. Endometrial Epithelial Cells Change Junctional Arrangement to Become Receptive 

It has been suggested that luminal and glandular endometrial epithelial cells lose polarity and convert to a more mesenchymal-like phenotype during the window of implantation [15,16]. This conversion is accompanied by fundamental changes of plasma membrane organization, which affects the membrane’s overall structure and composition and has been referred to as plasma membrane transformation [16,17]. The partial epithelial-to-mesenchymal transition presumably reduces the barrier function of the endometrium for the embryo to implant. Manifestations of the structural and functional transition in the human endometrium include the downregulation of N-cadherin [18], changes in apical marker enzyme expression [19,20], the reduction of apical microvilli [21] with the development of large apical membrane protrusions referred to as pinopodes [22], increased plasma membrane tortuosity [23], cytoskeletal reorganization [6,24,25], changes in cell–extracellular matrix contacts [26], and the rearrangement of the tripartite junctional complex [27,28] (Figure 3). 

**Figure 4:**
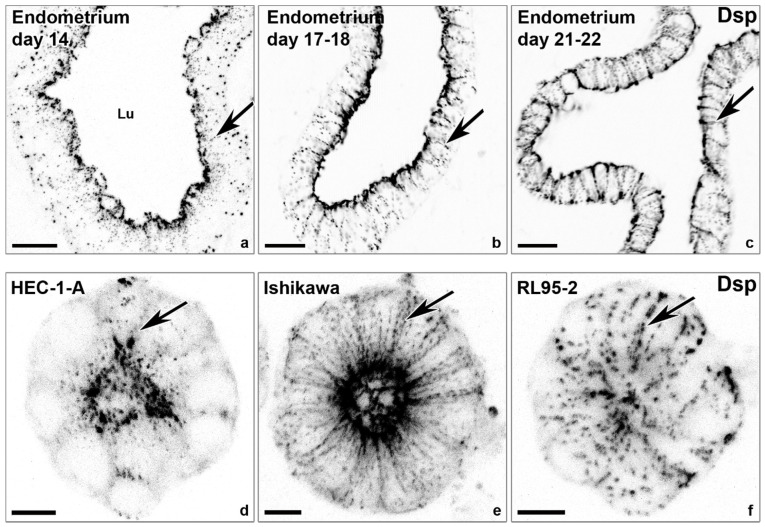
The lateralization of desmosomal cell–cell adhesions coincides with the window of implantation and polarization of endometrial epithelial cell lines. The confocal fluorescence micrographs (inverse presentation; single focal planes are shown in (**a**–**c**); the projections of 8 consecutive focal planes shown in (**d**–**f**)) reveal anti-desmoplakin (Dsp) reactivity that detects punctate desmosomes in the endometrial epithelial cell layer of the human endometrium obtained at different days of the menstrual cycle (**a**–**c**) and in gland-like spheroids (**d**–**f**) derived from endometrial adenocarcinoma cell lines with high polarity (HEC-1-A), intermediate polarity (Ishikawa), and low polarity (RL95-2). Note the different distributions of desmoplakin-positive desmosomes along the basolateral plasma membrane (arrows). Lu, lumen. Scale bars: 20 μm (**a**–**c**), 10 μm (**d**–**f**). The images were modified from [27,31].

The tripartite junctional complex consists, from apical to basal, of the circumferential tight junction, which tightly seals the intercellular space, the belt-like actin-associated adherens junction, and the spot-like keratin intermediate filament-associated desmosome. Desmosomal and adherens junction proteins are mainly localized subapically during the proliferative (days 6–14) and early-to-mid secretory phase (day 20) in endometrial epithelial cells of the stratum functionale. Both junctions become evenly distributed along the lateral plasma membrane during the window of implantation [27] (see also Figure 4). The gene expression of junctional proteins such as that of desmoplakin and E-cadherin, however, does not significantly change during the menstrual cycle [18,27]. Tight junctions remain apical [27] but develop additional strands that move further down along the lateral plasma membrane in luminal endometrial epithelial cells around the time of ovulation [29]. A potentially relevant observation in this context is that the GTPase Rab13, which has been implicated in endosomal trafficking to the lateral plasma membrane, colocalizes with the desmosomal cadherin desmoglein 2 during the window of implantation but not with the tight junction protein ZO-1 or the adherens junction protein E-cadherin in the endometrium of rats [30]. The authors therefore proposed that Rab13 contributes to the redistribution of desmosomes. Overall, the rearrangement of the junctional complex may weaken cell–cell adhesion and prepare the endometrium for trophoblast invasion. Comparable alterations of junctional rearrangements were also observed in 3D in vitro cell models [26] (see also Figure 4). Thus, a redistribution of desmosomes and adherens junctions was documented in response to estrogen, progesterone, and a combination of both in 3D acini prepared from the moderately polarized endometrial epithelial cell line Ishikawa [26].

Changes in cell–extracellular matrix contacts are also observed as intercellular spaces widen and extracellular matrix components intercalate between basolateral intercellular spaces. An indication of this re-organization and altered mechanical interface is the reported menstrual cycle-dependent redistribution of the hemidesmosomal α6/β4-integrin, which serves as a laminin-332 receptor. The lateralization of the a6-integrin subunit has been observed in vivo in a receptive endometrium and in vitro in endometrial epithelial cell-derived 3D acini upon hormone stimulation [26,32]. In addition, significant alterations in the expression pattern of α1β1, α4β1, αvβ3, and α6β1 integrins have been detected during the menstrual cycle and, more specifically, during the window of implantation in the endometrial epithelium [32,33]. 

Together, the findings indicate that the hormone-dependent changes in endometrial epithelial cells reflect a transition between two physical states that have been referred to as jamming and unjamming (see, e.g., [34,35]). Unjammed cells are free to move as in a fluid, whereas the movement of jammed cells is constrained as in a solid. The transition between both states depends on a variety of biophysical parameters such as intercellular adhesion, cortical tension, and 3D matrix confinement, and it is reflected by changes in cell shape. The underlying biomechanical concept is based on observations in epithelia that undergo fundamental changes in epithelial cohesiveness, tension, and stress regulating basic cellular properties such as migration, proliferation, and carcinogenesis (e.g., [36]). Future studies will reveal whether this concept also applies to menstrual cycle-dependent endometrial plasticity.

## 4. Hormone-Dependent Cytoskeletal Rearrangements Regulate Trophoblast Adhesion

It has been suggested that cytoskeletal rearrangements alter epithelial cell stability during the menstrual cycle [37,38,39,40,41]. In accordance, the inhibition of actin polymerization by cytochalasin D in the endometrial epithelial cell line RL95-2, which is commonly used as a model of a receptive endometrium [42,43], was found to reduce trophoblast-adhesiveness at the apical endometrial cell pole [24]. The researchers speculated that the treatment led to the dissociation of integrins and the actin cytoskeleton and that, conversely, an intact integrin-actin system is needed for trophoblast adhesion in non-polar RL95-2 cells. 

Even less is known about menstrual cycle-dependent changes in the organization of the cytoplasmic intermediate filament cytoskeleton of endometrial epithelial cells. However, the observed alterations in desmosome distribution and the actin system [24,26,27], which are known to act in concert, indicate that the 3D organization of the intermediate filament cytoskeleton is also modulated during the menstrual cycle. Following other polarized epithelia [44,45,46,47], we expect that the keratin network is apically concentrated below the actin-rich terminal web in a non-receptive endometrium and propose that this distribution switches to a non-polarized, pancytoplasmic distribution during the window of implantation. In addition to the structural re-organization of intermediate filament cytoskeleton, compositional alterations also occur. As such, keratin 13 was shown to be exclusively expressed in the surface epithelium during the proliferative phase and was not detected in a secretory-phase uterine epithelium [25]. Similarly, keratin 86 was found to be upregulated in a murine endometrium during peri-implantation [48]; furthermore, keratin 86 expression was enhanced by high estrogen levels and ameliorated by progesterone. 

## 5. Steroid Hormones Regulate Apicobasal Organization in Endometrial Epithelial Cells

Little is known about the activity of cellular pathways that may regulate polarity switching in the endometrial epithelium during the menstrual cycle. In polarized epithelial cells, apicobasal polarity is mainly controlled by the Crumbs, Par, and Scribble complexes, which were first described in *Drosophila melanogaster* [49]. Crumbs and Par specify the apical domain, and Scribble the basolateral domain. The immunostaining of the endometrium revealed that all three protein complexes are present in luminal and glandular epithelial cells, with limited localization in the stroma [50]. Steroid hormone-driven cyclic changes of these complexes in luminal endometrial epithelial cells of the stratum functionale were reported [50]. Atypical protein kinase C, which is part of the apically localized Par complex, was shown to be downregulated during the entire secretory phase [50]. It was further observed that Crumbs and the Crumbs-associated protein Stardust were downregulated during the late secretory phase [50]. These findings indicate a loss of apical polarity during the secretory phase and, especially, during the window of implantation, and they suggest that this may be regulated by the Par and the Crumbs complexes under the control of estrogen and progesterone. 

The basolateral determinant Scribble is also downregulated in luminal endometrial epithelial cells during mid and late secretory phase, but it is upregulated in stromal cells during the late secretory phase and could therefore play a role in decidualization [50]. The hormone-dependent regulation of Scribble was also reported in the endometrial cancer cell line ECC-1 [50], which was later reclassified as Ishikawa [51]. A combination of estrogen, progestin, and the trophoblast-derived hormone human choriogonadotropin (hCG) induced the downregulation of Scribble. Furthermore, the experimental downregulation of Scribble by siRNA increased trophoblast adhesion and improved decidualization efficiency [50]. 

In conclusion, it appears that apical polarity is controlled by maternal steroid hormones, whereas basolateral identity is affected by embryonic trophoblast-derived signals such as hCG.

## 6. Mechanical Stimuli Enhance Decidualization of Endometrial Stromal Cells

As discussed, luminal endometrial epithelial cells loosen their cell–cell adhesion, reorganize their cytoskeleton, and shift their morphology towards a mesenchymal phenotype during the secretory phase of the menstrual cycle in order to become receptive for embryo implantation by resembling partial epithelial-to-mesenchymal transition [15,50]. In contrast, the endometrial stromal cells undergo a progesterone-dependent mesenchymal–epithelial transition during decidualization [52,53]. Around menstrual cycle days 24–28, endometrial stromal cells differentiate from a fibroblastic to an epithelioid-like state [54]. Interestingly, the origin of this differentiation process is closely localized to the terminal spiral arterioles [9]. Using a novel organ-on-a-chip microfluidic system, it was shown that laminar shear stress enhances the decidualization of primary endometrium-derived stromal fibroblasts when co-cultured with endothelial cells [55]. This observation supports the idea that mechanical stimuli imposed by the increased blood flow of the differentiated endometrium contribute to the regulation of human decidualization. The colloidal probe nanoindentation of endometrial stromal cells detected cell softening during in vitro decidualization, which may be caused by cytoskeletal reorganization, specifically by the downregulation of vimentin, upregulation of keratins, and destabilization of F-actin [6,56]. Conversely, increased matrix stiffness was found to induce F-actin fiber formation and proliferation of endometrial stromal cells in vitro [57].

## 7. Mechanical Cues Modulate Embryo–Endometrial Interaction

To study the adhesion forces between trophoblast cells and endometrial epithelial cells, in vitro experiments were performed using human cell lines. Endometrial epithelial cell lines with either a high degree of polarization (HEC-1-A) or a low degree of polarization (RL95-2) were used to mimic non-receptive and receptive endometria, respectively [42,58,59,60,61]. They were combined with the human choriocarcinoma-derived trophoblast-type cell line JAR [62]. Time-dependent adhesion was measured with the help of an atomic force microscopy tip that was functionalized with JAR cells and brought into contact with HEC-1-A and RL95-2. After contact times of 20–40 min with a JAR-coated cantilever, the polarized HEC-1-A cells showed an adhesive force maximum of 16 ± 4 nN and a relatively smooth cell surface separation at a distance of 8–20 µm. On the other hand, the less polarized RL95-2 cells did not separate from JAR cells but ruptured at forces of 15 ± 4 nN at a distance of about 45 µm, indicating that cell–cell adhesion proteins were mechanically destroyed by the pulling [63]. In contrast to HEC-1-A, the apical plasma membrane of RL95-2 lacks microvilli [59] and has a thinner glycocalyx [61], thus enabling easier accessibility for trophoblast adhesion. 

Interestingly, firm adhesion between JAR and RL95-2 was only observed when the JAR-functionalized tip indented the apical surface of the RL95-2 cells [63]. This further highlights the idea that implantation is dependent on mechanical cues. In vivo support for this idea was revealed in a study showing that mammalian blastocysts undergo swelling during apposition just before implantation [64]. Thus, the hydrostatic pressure exerted by the embryo might play a role in causing the endometrium to become more receptive.

## 8. Trophoblast Penetration of the Epithelial Barrier Is a Multimodal Process

The penetration of endometrial epithelial cells by the embryonic trophoblast has not been observed in humans, and practically no histological data regarding the very initial stages of this process are available (regarding later stages, see, e.g., [65,66]). It is known, however, that the trophoblast can penetrate the epithelial barrier from both the apical side, which occurs after attachment to the apical side of the endometrial surface epithelium, and the basal side, which occurs during the erosion of the glandular epithelium after trophoblast invasion. Both processes may involve a combination of protease-mediated channeling and active movement that involves the contractile acto–myosin system. Furthermore, it is generally accepted that trophoblast cells migrate as both single cells (i.e., single extravillous trophoblast cells in the decidua) and cell collectives (i.e., villous trophoblast cells). It remains to be worked out, however, whether trophoblasts move transcellularly or through the weakened paracellular spaces of the endometrial epithelium, as well as which mechanophysical mechanisms guide the transepithelial migration of the trophoblast. Complex co-culture systems are being established to investigate mechanistic and molecular details of trophoblast migration cells across the endometrial epithelial cell layer. Our own attempts using human endometrial epithelial-cell-line-derived gland-like acini and human trophoblast cells have been encouraging, because they have revealed an inverse correlation between the degree of endometrial epithelial polarization and trophoblast invasion [31]. 

## 9. Invading Extravillous Trophoblast Cells Increase Decidual Stiffness by Remodeling Vasculature and Extracellular Matrix 

The uterus remodels to provide an optimal embryonic environment during gestation, especially regarding placentation, i.e., the development of the placenta. The placenta develops from the trophectoderm of the blastocyst during the first trimester of pregnancy. Highly invasive trophoblast cells form cell columns at the tips of anchoring villi, from which single extravillous trophoblast cells migrate into the decidua basalis. The invading trophoblast cells enter and remodel maternal spiral arteries and endometrial glands to ensure embryonic nutrition (Figure 2) [67,68,69]. 

To feed and support the developing embryo, the uterine blood supply is enhanced. Remarkably, maternal uterine blood flow increases manyfold during pregnancy [70]. In the non-pregnant state, uterine artery blood flow ranges from 22.4 ± 7.3 mL/min during the proliferative phase to 30.7 ± 13.7 mL/min in the secretory phase [71], whereas blood flow in the late phase of pregnancy reaches up to 750 mL/min [70]. It is safe to assume that the shear stress of the uterine arteries rises with the increase in blood volume. Arteries undergo extensive dilation, and spiral arteries are actively remodeled by extravillous trophoblast cells, changing not only their morphology and structure but also their mechanical properties. An ex vivo study performed by Abbas et al. in 2019 [72] measured the stiffness of the decidua basalis, decidua parietalis, and placenta at a gestational age of between 8 and 10 weeks by atomic force microscopy; they revealed that the decidua basalis with placental villi was significantly stiffer than the non-invaded decidua parietalis and placenta [72]. Hence, invading extravillous trophoblast cells mainly contribute to or induce endometrial stiffness. The remodeling of spiral arteries results in the replacement of the musculoelastic wall by a fibrinoid material that embeds intramural extravillous trophoblast cells [73]. Large areas within the invaded decidua become necrotic and accumulate fibrinoid material in Nitabuch’s layer, presumably increasing local tissue stiffness [74]. 

A second factor contributing to enhanced endometrial stiffness during embryo implantation and development is the remodeling of the extracellular matrix when invading trophoblast cells secrete extracellular matrix proteins and matrix metalloproteases. Trophoblast cells predominantly produce laminin, fibronectin, collagen IV, and fibrillin I [72,75,76]. Decidual cells also produce laminin and collagen IV [77]. Though laminin is the main extracellular matrix component of the villous trophoblast basement membrane, single extravillous trophoblast cells predominantly produce fibronectin [75]. Fibronectin is quite soft and assembles into highly extensible fibers when stress is applied [78]. Though fibronectin may be a key player in extravillous trophoblast migration [79], it is unlikely to be associated with decidua stiffening. Interestingly, collagen IV expression is significantly upregulated in the stroma of an invaded decidua basalis compared to a decidua parietalis, especially around invading extravillous trophoblast cells [76]. Due to collagen IV’s non-fibrillar nature, it is not directly associated with tissue stiffness; rather, it is associated with enhanced stress resistance [14]. The glycoprotein fibrillin I, on the other hand, arranges in microfibril networks that act as stiff polymers with a Young’s modulus between 78 and 96 MPa [80]. In 2019, Abbas et al. [72] hypothesized that a specific combination of fibrillin I-rich extracellular matrix proteins produced by extravillous trophoblast cells induces the stiffening of the decidua basalis while degrading stromal extracellular matrix proteins with serine proteases and matrix metalloproteases (see also [81,82]).

Conversely, connective tissue stiffness influences the morphology and invasion potential of extravillous trophoblast cells [83], which may serve as a negative feedback loop. When the extravillous trophoblast cells reach the myometrium, which is considerably stiffer than the decidua, they stop migrating altogether and fuse into multinucleated giant cells [84]. The function of these giant cells is not well understood, but they secrete collagenases and matrix metalloproteases, which may soften the maternal tissue [85]. 

## 10. Superficial Endometrial Injuries May Improve Embryo Implantation Rates

The mechanical injury of the endometrium by a uterine scratch biopsy during the early-to-mid secretory phase can improve implantation and birth rates in patients with recurrent implantation failure ([86,87]. Contrasting results [88,89] may be explained by differences in study design, selection of patients, patient age, or the number of biopsies performed. A recent systematic review and meta-analysis [90] of ten selected studies with 1260 patients revealed overall higher clinical pregnancy and live birth rates in the endometrial injury group.

Decidualization in rodents, which is normally triggered by the embryo, can be artificially and fully induced in pseudopregnant or hormonally-prepared animals with diverse mechanical stimuli such as oil injection [91]. Mechanical manipulation is linked to decidual cell growth, which is triggered by cytokine and growth factor secretion. Furthermore, microarray assays determined that genes that are known to be involved in the implantation process, including transmembrane protein mucin 1, phospholipase A2, and uroplakin Ib, are upregulated after uterine injury [92]. The cytokine prostaglandin E2 may play a major role in scratch-induced endometrial alterations because it is a known regulator of decidualization [93,94]. Interestingly, the epithelial mechanosensitive sodium channel (ENaC), which is synthesized in endometrial epithelial cells, regulates prostaglandin E2 production [95,96,97,98]. It was therefore hypothesized that ENaC is a key transducer for improved implantation rates after taking endometrial scratch biopsies [99]. This is supported by the detection of lower levels of endometrial ENaCα subunit expression in patients undergoing in vitro fertilization with failed pregnancy [98]. 

## 11. ENaC, Piezo1 and TRP Are Functionally Expressed in Endometrial Epithelial Cells and May Contribute to Embryo–Endometrial Epithelial Cell Crosstalk

It has been demonstrated by whole-cell patch-clamp experiments of human endometrial biopsies that the application of a mechanical stimulus (i.e., indentation) results in an increase in current density. This effect was fully inhibited by GsMTx4, an inhibitor of mechanosensitive ion channels [100,101]. Thus far, besides ENaC, Piezo1 and members of the transient receptor potential (TRP) superfamily have been described in the endometrium [97,101,102].

ENaC is located on the apical plasma membrane of human luminal endometrial epithelial cells [97]. When stimulated with the protease activator trypsin, a calcium influx in human endometrial epithelial cells has been observed [101,103], indicating the functional expression of this ion channel. As discussed above, ENaC may play a crucial role in responding to mechanical stimuli and may therefore be a critical protein in embryo–endometrium interaction during implantation. 

Piezo1 is a mechanically-activated ion channel that is known to affect cytoskeletal organization and function (review in [104]). Piezo1 was found to be functionally present in human EEC at different stages of the menstrual cycle [101]. The expression of Piezo1 but not Piezo2 was detected by RT-qPCR in human endometrial epithelial and stromal cells [101]. The mechanosensitivity of endometrial epithelial cells was shown by mechanically stimulating the cell membrane, which resulted in a robust calcium influx that could be intensified by the Piezo1 agonist Yoda1 [105]. 

It may be relevant for understanding the role of ENaC and piezo in embryo–endometrial crosstalk that they are themselves regulated by submembranous actin dynamics (review in [106]), which may be part of a regulatory feedback loop. The relevance of this fact is emphasized by the observation that reduced actin polymerization has been identified as a potential mechanism of viral-induced implantation failure [107].

Several members of the classical (C) and vanilloid (V) TRP family, which are produced in various vertebrate tissues, have been found to be activated by mechanical stimuli such as stretching (TRPV2, TRPV4, and TRPC1) or shear stress (TRPV4) (as reviewed in [108,109]). In non-pregnant endometrial biopsies, these and other members of the TRP channel protein family could be detected by qRT-PCR [101,102]. The expression levels of TRPV2, TRPV4, and TRPC1 vary during the menstrual cycle: TRPV2 is upregulated during the late secretory phase and menstruation, TRPV4 is downregulated during the entire secretory phase, and TRPC1 is downregulated during menstruation but does not show different expression levels during the proliferative and secretory phases [102]. These findings stress that TRP expression in the uterus is regulated by steroid hormones, as has been shown for progesterone-responsive TRPC5 [110] and estrogen-responsive TRPV6 [111]. Remarkably, these changes in functional relevance occur during the window of implantation [102], potentially responding to the physical forces that the embryo exerts toward the luminal endometrial epithelium and may therefore be critical for embryo invasion. However, the functional expression of TRPV2, TRPV4, and TRPC1 could not be detected in human endometrial epithelial cells by measuring cellular calcium influx when stimulated with an agonist [101]. Thus, the role of TRP proteins in the endometrium and during embryo implantation is still unclear. 

## 12. TAZ Protein Is Downregulated in Endometrial Stromal Cells during Decidualization

Intracellularly, the conversion of mechanical forces into biochemical signals takes place through the activation of several pathways. One of the most prominent pathways of mechanotransduction is the Hippo pathway and its downstream effectors YAP (Yes-associated protein) and TAZ (transcriptional coactivator with PDZ-binding motif), as reviewed in [112,113,114]. The immunostaining of the human uterine endometrium revealed specific YAP and TAZ reactivity in epithelial and stromal cells [115,116]. Additionally, YAP and TAZ could be detected by immunoblotting and immunohistochemistry in the endometrial cell line Ishikawa (erroneously referred to as ECC-1 in some publications) and in human stromal fibroblasts [117]. The TAZ protein is downregulated in endometrial stromal cells during the secretory phase. In glandular and luminal endometrial epithelial cells, there are no differences in TAZ expression patterns between the secretory and proliferative phases. During in vitro decidualization, which was induced by combined interleukin 1 beta and steroid hormone stimulation, TAZ protein levels but not mRNA levels were downregulated in stromal fibroblast cells that were obtained from human endometrial tissue [117]. Finally, the reported increased YAP protein levels in decidual endometrial stromal cells during early pregnancy [118] can be taken as a hint that the invading embryo regulates uterine Hippo pathway. 

## 13. The Endometrium Stiffens during Pregnancy

Standardized methods to measure the mechanical properties of the human reproductive female tract in vivo are virtually non-existent [119]. Information on uterine mechanics is therefore scarce and has mostly been limited to analyses of the myometrium. Ex vivo studies have shown that a non-pregnant, non-contracting myometrium has non-linear material properties and is stiffer under tension than under compression [120,121]. The material behavior is not influenced by the menstrual cycle phase or age of the patient [120]. More recent aspiration and compression tests of entire human uteri confirmed their anisotropic and viscoelastic properties [122,123,124]. 

Only as recently as 2019, Abbas et al. [72] were the first to investigate the tissue stiffness of a human endometrium in the non-pregnant secretory phase in comparison to first trimester decidua and placenta. Using atomic force microscopy, they measured an apparent elastic modulus of 250 Pa in ex vivo endometrial tissue samples from non-pregnant women, whereas the elastic modulus of ex vivo tissue samples from the decidua basalis, i.e., the endometrial site of placental invasion, was determined to be 1000 Pa [72]. 

## 14. Conclusions

Within the last few years, novel and exciting research approaches have emphasized that researchers studying the menstrual cycle and human implantation must consider the impact of mechanics. With novel technologies becoming available, it will be possible to investigate aspects of female reproductive tract biomechanics in humans, thus heralding an end to the difficult-to-interpret, highly variable, and small datasets of the past. They may also provide an inroad into the analysis of specimens that are confounded by pathologies, such as myoma, sarcoma, or uterine prolapse, that may affect tissue mechanics, as has been observed in endometriosis, an estrogen-dependent disorder that is characterized by the presence of endometrial tissue outside the uterus [125]. Eutopic endometrial stromal cells from healthy women are stiffer and have a lower deformation index than cells from endometriosis patients when placed inside a microchannel system [126].

To conclude, the role of mechanical forces during the menstrual cycle and the mechanical interplay of the embryo and endometrium is far from understood at the cellular and molecular levels. To uncover the molecular mechanisms that determine the mechanical cross-talk between the developing human embryo and endometrium during implantation, in vitro systems must be further developed (see the recent review in [127]). These approaches may involve novel 3D co-culture systems (e.g., [128]) and hormone-sensitive endometrial organoids (e.g., [129,130,131]; see recent reviews in [132,133]).

## Data Availability

Not applicable.

## Glossary

Atomic force microscopy (AFM)	Method used in mechanobiology to quantify and apply mechanical forces in biological systems and biomaterials. The tip of a cantilever is used to probe and indent the underlying surface.
Biomechanics	Approach to describe the structural properties of cells, tissues, organs and organisms by mechanical concepts of engineering sciences.
Complex shear modulus |G *|	Response of viscoelastic material to vibration, expressed in Pa (=N/m²).
Compression	Application of a mechanical force that results in a contraction of the material.
Elasticity	Ability of a material to resume its normal shape after being stretched or compressed. It can be quantified, for example, by Young’s modulus “E”.
Mechanical force	A force that involves contact with another object and that produces a change in state of rest or motion. Mechanical forces are expressed in Newton (N = kg m/sec²).
Mechanobiology	Interdisciplinary field that studies the effects of mechanical forces on the physiology and pathology of living systems.
Mechanosensing	Ability of cells to sense and respond to mechanical stimuli.
Mechanosensitivity	Quantifiable ability of a cell to sense and respond to a specific mechanical stimulus.
Mechanotransduction	The molecular conversion of a mechanical signal into a biological cell response.
Mechanotransmission	The transmission of an applied mechanical load to specialized structures.
Nanoindentation	Technique to measure the hardness of a material by pressing a probe against a sample.
Shear stress “τ”	Stress that is applied parallel or tangential to the surface of a material.
Stiffness	Technical term to describe the elastic properties of a material. It can be quantified by Young’s modulus “E”.
Strain “ε”	Measure of material deformation as a result of tension or compression expressed in percentage.
Stress	Force per unit area within materials that arises from externally applied forces. It is measured in Pascal (Pa = N/m²).
Tension	The application of a pulling force that results in an elongation of the material.
Young’s modulus “E”	Stiffness value expressed in Pa (=N/m²). It describes the elastic properties of a solid undergoing tension or compression in only one direction (E = σ/ε).
Viscoelasticity	Property of materials that display a mixture of viscous and elastic behavior when stressed.

## Appendix A

**Info Box 1—The Uterine Wall (See Also Figure 1)**

The uterine wall is composed of three main layers; from outside to inside, they are: (*i*) the loose connective tissue-containing perimetrium, which is covered by mesothelium towards the abdominal cavity, (*ii*) the smooth muscle cell-containing contractile myometrium, and (*iii*) the composite and highly variable endometrium. The endometrium consists of an epithelial compartment and a connective tissue compartment. The simple epithelium lines the endometrial surface and is contiguous with the tubular uterine glands. The connective tissue consists of stromal fibroblasts, blood vessels, and a variable number of immune cells. During the estrogen-driven proliferative phase of the menstrual cycle (days 6–14), uterine glands and the surrounding stroma enlarge to build up the stratum functionale. With ovulation and the subsequent rise in progesterone, endometrial growth comes to a halt and the endometrium prepares for nidation during the secretory phase (days 15–28). The secretory phase encompasses the differentiation of the endometrial epithelium, the secretion of uterine glands, and the decidual reaction, i.e., mesenchymal-to-epithelial transition of stromal fibroblasts, vascular remodeling, and an increase in leukocytes. Embryo implantation typically occurs midway through the secretory phase. If implantation does not take place, the stratum functionale is shed during menstruation (desquamation; days 1–5), leaving behind a residual endometrium only consisting of a remnant stratum basale, which then enters another hormone-instructed menstrual cycle.

**Info Box 2—The Endometrial Epithelium and Underlying Connective Tissue (See Also Figure 3)**

The endometrial epithelium is a typical polarized epithelium. The apical surface is directed towards the air/liquid-filled uterine lumen and contains microvilli and cilia. It is separated from the basolateral surface by a junctional complex consisting of the polarity-maintaining zonula occludens (tight junction), the actin filament-linked zonula adherens (adherens junction), and the keratin intermediate filament-linked macula adherens (desmosome) from top to bottom. The epithelial cells are anchored to the underlying basement membrane. The epithelial cells become receptive for implantation during the secretory phase. The cell shape changes from columnar to cuboidal; pinopodes, i.e., specialized protrusions, appear at the cell surface; and apical markers are lost. Furthermore, the glandular epithelial cells develop glycogen vacuoles and increase their secretion.

The apolar stromal cells underneath the endometrial epithelium are embedded in the extracellular matrix. They have migratory and invasive characteristics. During the secretory phase, they pre-decidualize by increasing their cytoplasmic volume and increasing the extracellular matrix volume. In the absence of a conceptus, falling progesterone levels lead to tissue breakdown catalyzed by leukocytes, which secrete matrix metalloproteases. The myometrial contractions and vasoconstriction of spiral arteries then lead to a cleavage between the stratum basale and stratum functionale.

**Info Box 3—Human Embryo Implantation (See Also Figure 2)**

After fertilization, the embryo moves from the Fallopian tube to the uterus, reaching it around days four-to-five as a blastocyst. The blastocyst is a fluid-filled sphere that is made up of 200–300 cells. It consists of an outer trophoblast cell layer that gives rise to the placenta and an inner cell mass (embryoblast) that develops into the embryo. The endometrium presents an efficient barrier that can only be penetrated by the embryo during a limited period of the menstrual cycle, i.e., during the window of implantation (days 19–23; [134]). During the window of implantation, endometrial epithelial cells rearrange their junctions through the lateralization of desmosomes and adherens junctions. Implantation starts after the hatching of the blastocyst from the surrounding zona pellucida with apposition of the trophoblast to the luminal endometrial epithelial cells. The binding of trophoblast adhesion molecules to endometrial epithelial cell ligands results in the attachment of the blastocyst. The interaction of trophoblast L-selectin with oligosaccharides of endometrial epithelial cell pinopodes initiates adhesion and integrins, and cadherins are also likely involved [135,136]. The modes of trophoblast migration through the epithelial cell layer are not known. As the final step of implantation, trophoblast cells invade the decidual stroma to form the placenta, which provides nourishment for the embryo. Placental villi are formed, and the maternal stroma is remodeled by various matrix metalloproteases, serine proteases, and collagenases. Extravillous trophoblast cells migrate through the stroma and invade glands and spiral arteries, resulting in histiotrophic and hemotrophic nutrition of the embryo [68,69,137].

**Info Box 4—Mechanobiology in Health and Disease**

Mechanobiology is a highly interdisciplinary field that studies the effects of mechanical forces on the physiology and pathology of living systems. These mechanical forces include tension, compression, and shear flow, and they are modulated by cellular viscoelasticity and matrix rigidity (stiffness). Young’s modulus “E” provides a quantitative measure for these material properties. Cells perceive and respond to mechanical forces and extracellular matrix stiffness in a process referred to as mechanosensing. Mechanotransmission is defined as the transmission of an applied mechanical load to specialized structures. They include cell–extracellular matrix contacts [138,139] and, in closely spaced cells such as epithelial cells, cell–cell junctions [140]. Internal forces exerted by the cytoskeleton are also important, notably during cell migration. Mechanotransduction describes the cellular mechanism by which cells translate mechanical stimuli into biochemical signals, which elicit responses regulating, e.g., differentiation, proliferation, and apoptosis [141,142,143]. Healthy cells and tissues present viscoelastic behavior when stressed [144]. These responses are important for proper development and wound healing. Excessive mechanical stimulation, however, can result in pathologies, and many diseases are associated with alterations in cell stiffness. For instance, cancer cells are softer and have lower viscoelastic parameters than healthy cells [145]. Conversely, fibrosis is associated with increased tissue stiffness [146,147].
